# Mannosylated Linear and Cyclic Single Amino Acid Mutant Peptides Using a Small 10 Amino Acid Linker Constitute Promising Candidates Against Multiple Sclerosis

**DOI:** 10.3389/fimmu.2015.00136

**Published:** 2015-04-07

**Authors:** Stephanie Day, Theodore Tselios, Maria-Eleni Androutsou, Anthi Tapeinou, Irene Frilligou, Lily Stojanovska, John Matsoukas, Vasso Apostolopoulos

**Affiliations:** ^1^Immunology and Vaccine Laboratory, Burnet Institute, Melbourne, VIC, Australia; ^2^Department of Chemistry, University of Patras, Patras, Greece; ^3^Eldrug S.A., Patras, Greece; ^4^Centre for Chronic Disease, College of Health and Biomedicine, Victoria University, Melbourne, VIC, Australia

**Keywords:** multiple sclerosis, mannan, myelin basic protein, MBP_83–99_, altered peptide ligands

## Abstract

Multiple sclerosis (MS) is a serious autoimmune demyelinating disease leading to loss of neurological function. The design and synthesis of various altered peptide ligands of immunodominant epitopes of myelin proteins to alter the autoimmune response, is a promising therapeutic approach for MS. In this study, linear and cyclic peptide analogs based on the myelin basic protein 83–99 (MBP_83–99_) immunodominant epitope conjugated to reduced mannan via the (KG)_5_ and keyhole limpet hemocyanin (KLH) bridge, respectively, were evaluated for their biological/immunological profiles in SJL/J mice. Of all the peptide analogs tested, linear MBP_83–99_(F^91^) and linear MBP_83–99_(Y^91^) conjugated to reduced mannan via a (KG)_5_ linker and cyclic MBP_83–99_(F^91^) conjugated to reduce mannan via KLH linker, yielded the best immunological profile and constitute novel candidates for further immunotherapeutic studies against MS in animal models and in human clinical trials.

## Introduction

Multiple sclerosis (MS) is often a slowly progressive and chronic auto-immunologically mediated disease of the central nervous system (CNS), with inflammation around the myelin sheath (1–3). MS is primarily a T-helper 1 (Th1)-mediated disease, although Th17 cells also play a crucial role (4). Experimental autoimmune encephalomyelitis (EAE) is a commonly used experimental model of MS and represents an invaluable *in vivo* model for the evaluation of new immunotherapeutic approaches against MS. There is clear presence of autoreactive-T cells, which recognize encephalitogenic epitopes of myelin proteins, such as myelin basic protein (MBP), myelin oligodendrocyte glycoprotein (MOG), and proteolipid protein (PLP). These auto reactive T cells and their secretion primarily of Th1 cytokines (IFN-gamma) play a pathogenic role in the induction of disease. One promising immunotherapeutic approach against MS, involves the design and use of mutated peptides of immunodominant myelin epitopes to divert Th1 pro-inflammatory cytokines to an anti-inflammatory state to induce T cell tolerance. Studies have shown that T cell responses in patients are associated with the recognition of the 81–105 region of MBP (Q^81^DENPVVHFFKNIVTPRTPPPSQGK^105^), and with highest affinity and binding to HLA-DR2 (DRA, DRB1*1501) for MBP_83–99_ (E^83^NPVVHFFKNIVTPRTP^99^) (5–10). Although T cells from healthy individuals also recognize MBP_83–99_ the precursor frequencies are relatively low. The binding of MBP_83–99_ to HLA-DR2 is via hydrophobic V^87^ and F^90^ residues, and, V^86^, H^88^, F^89^, and K^91^ being TCR contact residues (10–15). Residue P^96^ is also a TCR contact site based on the crystal structure of HLA-DR2α-MBP_89–101_ complex (16). In a human phase II clinical trial in MS patients, substitution of the epitope MBP_83–99_, with several d-amino acids or Ala at the N terminal (NBI-5788, CGP77116) induced strong IL-5 and IL-13 cytokine responses; however, many patients developed dangerous side effects and the clinical trials were stopped (17, 18). These results indicated that further pre-clinical testing is required and new modified peptides need to be designed together with appropriate immunomodulatory adjuvants or carriers, for the peptide based immunotherapeutic approaches against MS.

Mannan (a poly mannose), which targets the mannose receptor expressed on dendritic cells and macrophages, has been used to target antigens to dendritic cells to stimulate appropriate immune responses. Mannan targets antigens to the mannose receptor for efficient uptake and presentation for T cell stimulation and also activates dendritic cells via toll-like receptor-4 (19). Whether mannan is conjugated to proteins or peptides in its oxidized (comprising aldehydes) or reduced (aldehydes reduced to alcohols) form, both bind to the mannose receptor efficiently; however, the stimulation of cytokines secreted by dendritic cells varies considerably, with reduced mannan inducing Th2 cytokines and oxidized mannan inducing Th1 cytokines (20). In *in vivo* studies, mice immunized with oxidized mannan-MUC1 (a tumor associated antigen) fusion protein are protected against the challenge of MUC1 expressing tumors, and in established tumors, oxidized mannan-MUC1 is able to reverse established tumors (21, 22). Similarly, in MUC1 transgenic mice oxidized mannan-MUC1 was immunogenic (23). Either a Th1 response (IL-2, IFN-gamma, IL-12, TNF-alpha, and IgG2a antibodies) or Th2 response (IL-4, IL-10, and TGF-beta and IgG1 antibodies) is induced depending on mode of conjugation, oxidized or reduced mannan (21, 22, 24). Other cytokines (IL-5, IL-6, IL-13, IL-15, and IL-18) are also secreted with either oxidized or reduced-mannan conjugates (24–26). In addition to Th1/Th2 type responses to MUC1 in mice, similar responses have been demonstrated in humans (27) and monkeys (28) with MUC1 protein and to an *Anaplasma marginale* MSP-1 peptide in cows (29). Since reduced mannan has the ability to induce Th2 responses, it was conceivable to use reduced-mannan conjugated to MBP peptides, with the idea being that Th2 cytokines would divert the Th1 cytokines in MS. Hence, the use of altered peptide ligands (APLs) to alter responses of the wild type peptide, in combination with reduced mannan constitutes a novel strategy for the immunotherapy of MS.

We previously demonstrated that linear and cyclic substituted APLs based on MBP_83–99_ and MBP_87–99_ epitopes, with single or double mutations at positions 91 and 96 as being the crucial TCR contact residues, conjugated to reduced mannan via the keyhole limpet hemocyanin (KLH) as a linker diverts the immune response from Th1 to Th2 enhancing the induction of anti-inflammatory cytokines IL-4 and IL-10 (30–35). In these studies, the cyclic and linear APLs were conjugated to KLH followed by conjugation to oxidized mannan and followed by its reduction to result in reduced-mannan conjugates. KLH was used as a linker between the peptide analogs and mannan as it is known that KLH has the ability to stimulate/enhance immunity (30–34), especially the induction of antibody responses. It was shown that antibodies were highly induced some of which did or did not cross react with the native peptide (30–34). KLH is a large molecular weight protein and the conjugate is highly heterogeneous and for future commercialization prospects following human clinical trials, would be highly complicated. In order to design a more precise immunotherapeutic with a smaller bridge, between peptide and reduced mannan, linear mutated MBP_83–99_ peptide analogs, with specific TCR substitutions, were conjugated to reduced mannan via a 10 amino acid bridge, [LysGly]_5_ [(KG)_5_] at the N-terminus of each peptide. In addition, the use of a smaller bridge like (KG)_5_ rather than KLH could potentially reduce antibody responses against the immunizing peptide, which could otherwise be a problem in human clinical trials. In this respect, a number of APL analogs with (KG)_5_ at its N-terminus and conjugated to reduced mannan were studied in SJL/J mice for their ability to induce immune responses, especially, T cell proliferation, IFN-gamma, IL-4, and IL-10 cytokine secretion and antibody responses.

## Results and Discussion

**Figure 1:**
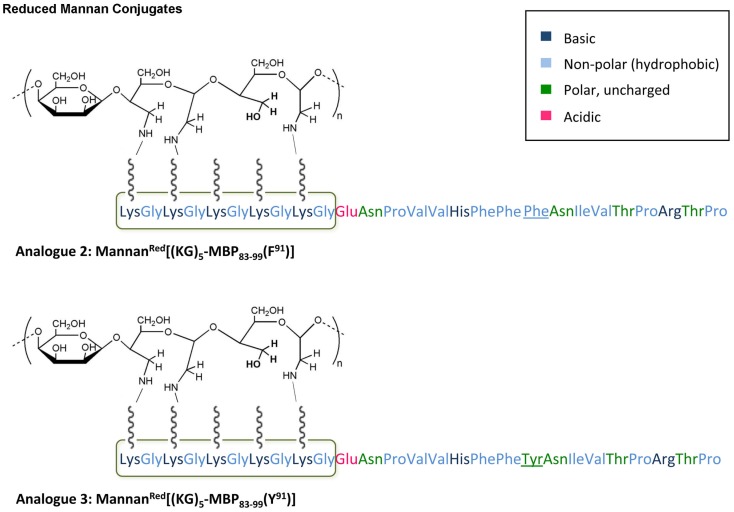
**Schematic presentation of analog 2: Mannan^Red^[(KG)_5_-MBP_83–99_(F^91^)] and analog 3: Mannan^Red^[(KG)_5_-MBP_83–99_(Y^91^)]**. The polarity of each amino acid is colory presented.

In this study, we have designed and synthesized mutant peptide analogs based on the 83–99 epitope of MBP, replacing the Lys at position 91 with a hydrophobic and aromatic amino acid (Phe or Tyr) or Val, His at positions 86 and 88 with Ala (Table 1). It is known that Val^88^ and Lys^91^ play a pivotal role in the interaction with the TCR and the activation of encephalitogenic T cells that are responsible for EAE and MS induction. The (KG)_5_ bridge was used in the N terminal of each linear peptide analog for conjugation with reduced mannan (Table 1; Figure 1). In our previous studies, the use of KLH bridge-mannan conjugates was less stable compared with reduced-mannan conjugation via (KG)_5_ bridge. Two cyclic analogs were also synthesized with head-to-tail cyclization and mutation at position 91 (Phe or Tyr). The cyclic peptide analogs **6**, **7** were conjugated with mannan via the KLH linker. It is known that the cyclic analogs are more stable in proteolytic degradation compared to linear counterparts (36). The backbone cyclization of peptides has been demonstrated to improve biological activity, *in vivo* stability and to reduce conformation freedom (37).

**Table 1:** **Summary of T cell proliferative, cytokine, and antibody responses**.

MBP_83–99_-(KG)_5_-peptide analogs-reduced mannan	T cell proliferation	ELISpot			Antibody responses
		IFN-γ	IL-4	IL-10	
**1** Mannan^Red^[(KG)_5_-MBP_83–99_]	+++	+++	−	−	−
**2** Mannan^Red^[(KG)_5_-MBP_83–99_(F^91^)]	+	−	++	+	−
**3** Mannan^Red^[(KG)_5_-MBP_83–99_(Y^91^)]	+	−	++	+	−
**4** Mannan^Red^[(KG)_5_-MBP_83–99_(A^88^)]	++	++	−	−	−
**5** Mannan^Red^[(KG)_5_-MBP_83–99_(A^86^)]	++	++	−	−	−
**Cyclic MBP_83–99_ peptide analogs-KLH-reduced mannan**	
**6** Cyclo(83–99)MBP_83–99_(F^91^)	+	−	++	+	−
**7** Cyclo(83–99)MBP_83–99_(Y^91^)	−	+++	+	−	−

*+++, very strong; ++, intermediate; +, weak; −, negative*.

**Figure 2:**
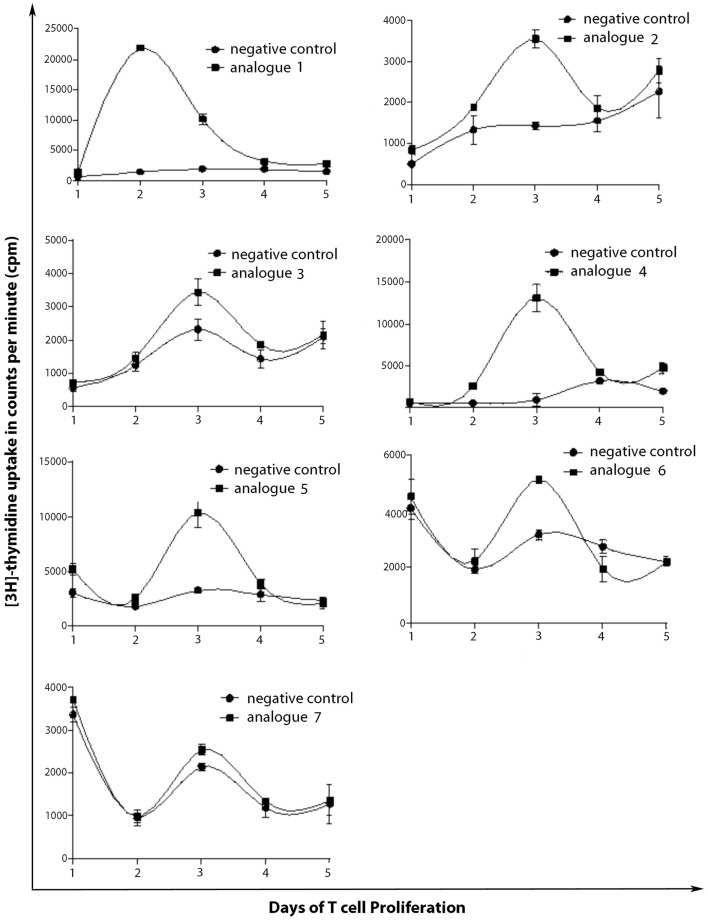
**T cell proliferation of spleen cells from immunized mice**. SJL/J mice were immunized using linear MBP_83–99_ analogs conjugated to reduced mannan via (KG)_5_ bridge (analogs **1–5**) and cyclic MBP_83–99_ analogs conjugated to reduced mannan via KLH linker (analogs **6** and **7**). ConA (internal positive control) yielded proliferation of more than 90,000 cpm and was excluded from the figures and no peptide (cells alone) was used as background negative control.

SJL/J mice (I–A^s^) is commonly used for the evaluation of MBP peptide analogs, as both murine I–A^s^ and human HLA-DR2 bind to MBP_83–99_ peptides. The synthesized MBP_83–99_-(KG)_5_ analogs conjugated to reduced mannan were used to immunize SJL/J mice. Immunized mice with analog **1**, induced very strong T cell proliferative responses after recall peptide *in vitro*. Peak proliferation was on day 2, which declined by day 3 (*p* < 0.001), and were background levels by day 4. Analogs **4** and **5** induced intermediate T cell proliferative responses (*p* < 0.001). Analogs **2**, **3**, and **6**, induced weak T cell proliferative responses, which were specific and significantly above background levels (*p* < 0.001). Peptide **7** did not stimulate T cell responses in SJL/J mice (Table 1; Figure 2).

**Figure 3:**
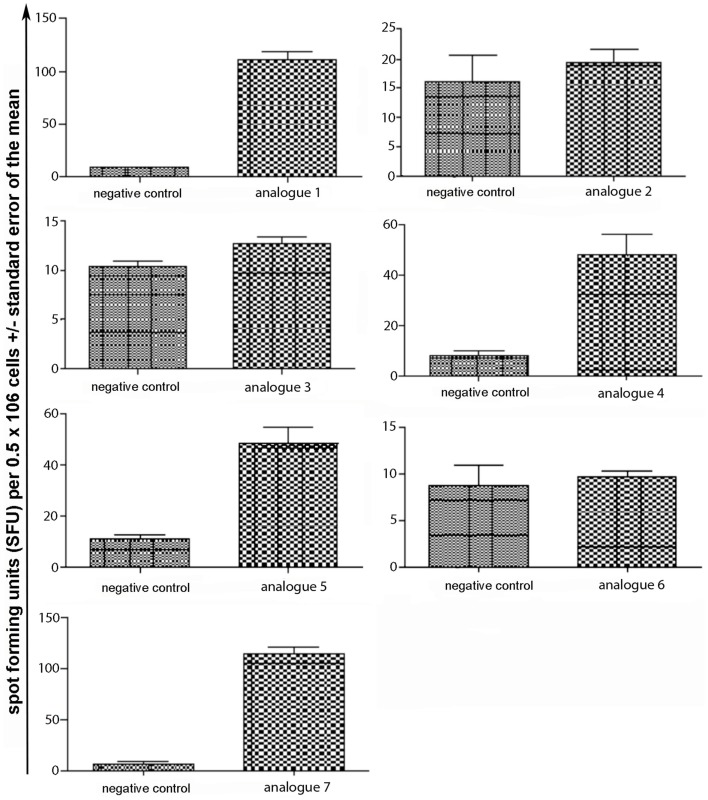
**IFN-gamma ELISpot assay of spleen cells from immunized mice**. SJL/J mice were immunized using linear MBP_83–99_ analogs conjugated to reduced mannan via (KG)_5_ bridge (analogs **1–5**) and cyclic MBP_83–99_ analogs conjugated to reduced mannan via KLH linker (analogs **6** and **7**). No peptide (cells alone) was used as background negative control. ConA was used as an internal positive control to ensure cells were reactive, not shown.

**Figure 4:**
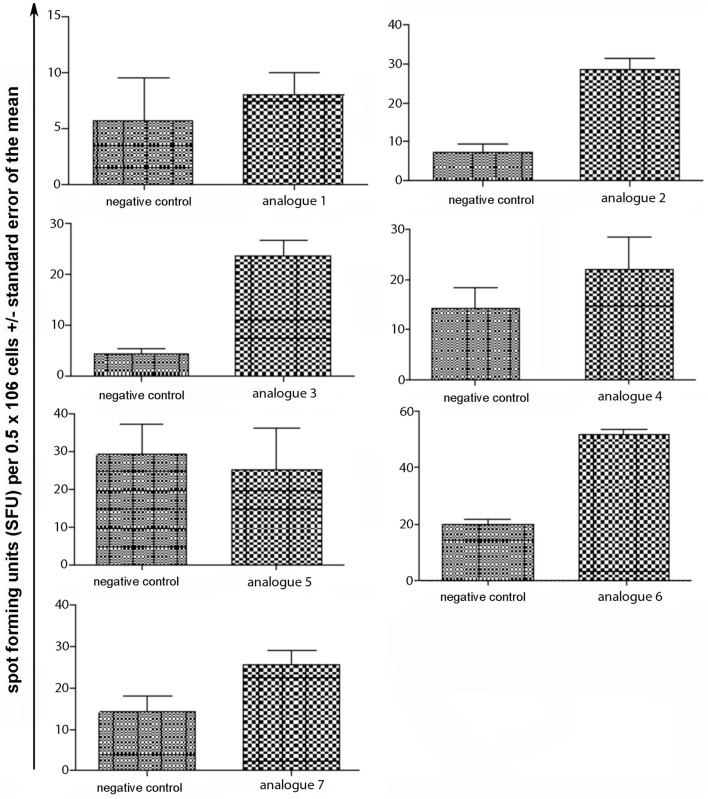
**IL-4 ELISpot assay of spleen cells from immunized mice**. SJL/J mice were immunized using linear MBP_83–99_ analogs conjugated to reduced mannan via (KG)_5_ bridge (analogs **1–5**) and cyclic MBP_83–99_ analogs conjugated to reduced mannan via KLH linker (analogs **6** and **7**). No peptide (cells alone) was used as background negative control. ConA was used as an internal positive control to ensure cells were reactive, not shown.

**Figure 5:**
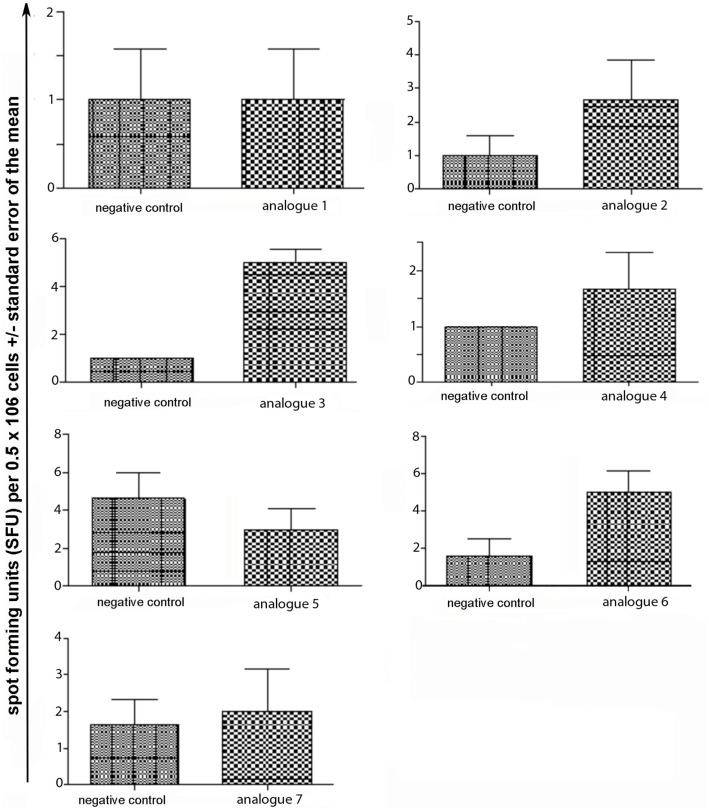
**IL-10 ELISpot assay of spleen cells from immunized mice**. SJL/J mice were immunized using linear MBP_83–99_ analogs conjugated to reduced mannan via (KG)_5_ bridge (analogs **1–5**) and cyclic MBP_83–99_ analogs conjugated to reduced mannan via KLH linker (analogs **6** and **7**). No peptide (cells alone) was used as background negative control. ConA was used as an internal positive control to ensure cells were reactive, not shown.

Spleen cells were isolated and assessed for T cell cytokine production (IFN-gamma, IL-4, IL-10) using the ELISpot assay (Table 1; Figures 3–5). Immunized mice with analogs **1** and **7** induced very strong IFN-gamma cytokine responses after recall peptide *in vitro* (*p* < 0.001). Analogs **4** and **5** induced intermediate IFN-gamma cytokine responses (*p* < 0.001). Analogs **2**, **3**, and **6** did not stimulate IFN-gamma cytokine responses in SJL/J mice (Figure 3). Immunized mice with analogs **2**, **3**, and **6** induced intermediate IL-4 cytokine responses (*p* < 0.001). Analog **7** induced weak IL-4 cytokine response, which was significant above background. Analogs **1**, **4**, and **5** did not stimulate IL-4 cytokine responses in SJL/J mice (Figure 4). Moreover, analogs **2**, **3**, and **6** induced very weak IL-10 cytokine response, which were significant above background (*p* < 0.05). All other peptides did not stimulate IL-4 cytokine responses in SJL/J mice (Figure 5). Interestingly, none of the peptide analogs generated antibody responses as measured by reactivity to native peptide and to peptide analog, from serum from immunized mice (Table 1).

## Conclusion

In previous studies, we used KLH as a linker between mannan and linear peptide. However, in an attempt to develop more defined conjugates for clinical use, we used a small 10 amino acid bridge (KG)_5_, to link mannan to linear APL. In this regard, the mannan^Red^[(KG)_5_-MBP_83–99_] analog (native epitope) induced very strong T cell proliferative and IFN-gamma cytokine secretion responses. The mannan^Red^[(KG)_5_-MBP_83–99_(F^91^)] and cyclic MBP_83–99_(F^91^) did not induce IFN-gamma responses and antibodies but induced IL-4 and IL-10 cytokines. The mannan^Red^ [(KG)_5_-MBP_83–99_(Y^91^)] did not induce IFN-gamma responses and antibodies but induced IL-4 and IL-10 cytokine; however, the cyclic counterpart cyclo(83–99)MBP_83–99_(Y^91^) generated strong IFN-γ cytokine the same as for the mannan^Red^[(KG)_5_-MBP_83–99_(A^88^)] and mannan^Red^[(KG)_5_-MBP_83–99_(A^86^)]. None of the peptide analogs generated antibody responses. Of note, here we used KLH as a linker between mannan and cyclic peptide, as (KG)_5_ bridge would not allow conjugation in the cyclic peptide form. In conclusion, the analogs **1**, **2**, and **6**, mannan^Red^[(KG)_5_-MBP_83–99_(F^91^)], cyclic MBP_83–99_(F^91^), and linear mannan^Red^[(KG)_5_-MBP_83–99_(Y^91^)], respectively, yielded the best immunological profile (Table 1) and are the most promising candidates for further studies *in vivo* in EAE studies, in humanized mice and *in vitro* using human T cell clones specific for MBP_83–99_ immunodominant epitope. Similar to our previous studies, using KLH as a linker between mannan and linear peptides, the more defined (KG)_5_ bridge presented here, induce specific immune responses. These peptides constitute novel analogs for further immunotherapeutic studies in animal models and possibly in human clinical trials.

## Experimental Procedures

### Solid phase peptide synthesis of linear and cyclic analogs

**Scheme 1:**
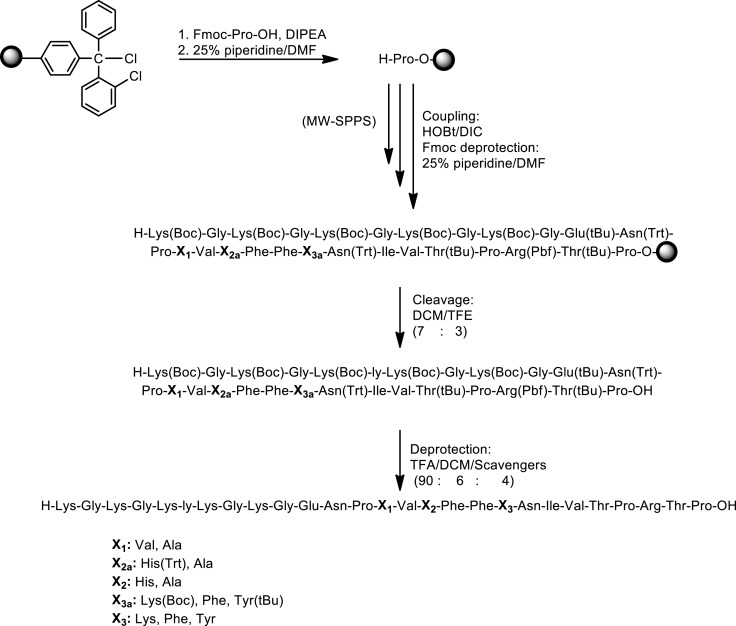
**Synthetic procedure of linear peptide analogs based on the MBP_83–99_ immunodominant epitope**.

**Scheme 2:**
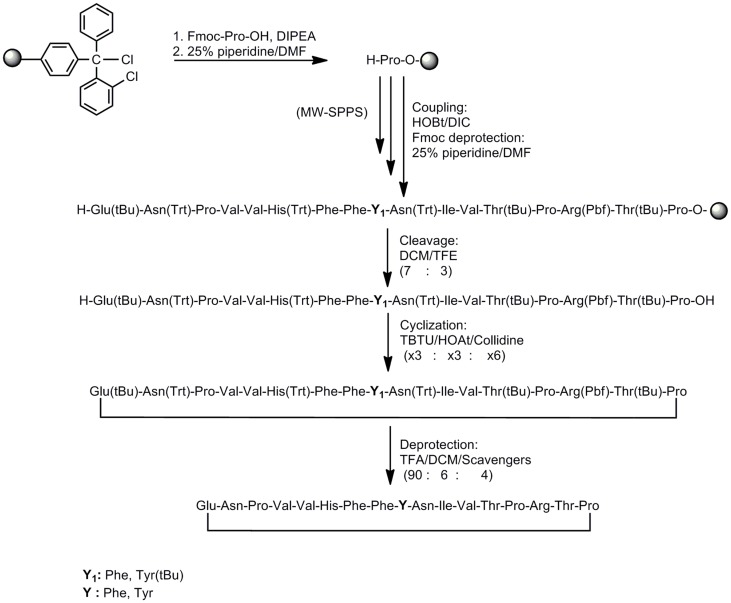
**Synthetic procedure of cyclic peptide analogs based on the MBP_83–99_ immunodominant epitope**.

Peptides (Table 1) were synthesized under microwave irradiation conditions (38, 39), following the Fmoc/tBu methodology, using the standard the *N*,*N*′-diisopropyl-carbodiimide (DIC) and 1-hydroxybenzotriazol (HOBt) as coupling reagents (40–43). The head-to-tail cyclization of analogs **6** and **7** was achieved using *O*-benzotriazol-1-yl-*N*,*N*,*N*′,*N*′-tetramethyluronium tetrafluoro borate/1-hydroxy 7-azabenzotriazol (TBTU/HOAt) and 2,4,6-collidine in dry dimethyformamide (DMF) (Schemes 1 and 2) (36, 41–45). The final peptides were further purified using semi-preparative reverse phase high performance liquid chromatography (RP-HPLC). The purity of peptides was higher than 98% as determined by analytical RP-HPLC and their identification was achieved by electron spray ionization mass spectrometry (ESI-MS).

### Conjugation of reduced mannan to linear and cyclic MBP_83–99_ peptide analogs

The conjugation between peptide to reduced mannan was achieved following a protocol earlier described for protein or peptide-KLH conjugations (21, 22, 31, 34). Briefly, 14 mg mannan (poly mannose from *Saccharomyces cerevisiae*, Sigma-Aldrich Ltd., was dissolved in 1 ml phosphate buffer, pH 6.0, and oxidized to polyaldehyde by treating with sodium periodate. Ethanediol was then added to the mixture and the mixture was passed through a PD-10 column (Sephadex G-25 M column, Pharmacia Biotech., Sweden) equilibrated with 0.1 M bicarbonate buffer pH 9.0 and the oxidized mannan fraction collected. Conjugation of linear peptide analogs-(KG)_5_ (analogs **1–5**) and cyclic analogs-KLH (analogs **6** and **7**) to oxidized mannan was performed in bicarbonate buffer, pH 9.0, in dark (Table 1; Figure 1). The addition of sodium borohydride for 6 h at room temperature resulted in reduced-mannan conjugates (46). The final MBP peptide analogs conjugated with mannan were analyzed by SDS PAGE (44).

### Mice and immunizations

Female 6- to 8-week-old SJL/J mice, used in all experiments, were purchased from Walter and Eliza Hall Institute (VIC, Australia) and housed at the Biological Research Laboratory at Burnet Institute (Austin Campus), Heidelberg, VIC, Australia. SJL/J mice were immunized with 50 μg of each peptide analog (Table 1) conjugated with reduced mannan, twice on days 0 and 14, intradermally (at the base of the tail). All studies were reviewed and approved by Austin Health and Alfred Health animal ethics committee.

### Immunological assays

#### ELISpot

Spleen cells from immunized SJL/J mice were isolated 14 days after the last immunization and assessed by ELISpot for IFN-γ, IL-4, and IL-10 secretion by T cells. IFN-γ ELISpot assay was performed on MultiScreen-IP Filter Plate (MAIP S4510) with hydrophobic PVDF filters (Millipore, UK), while IL-4 and IL-10 ELISpot assays were performed on MultiScreen-HA Filter Plate (MAHA S4510) with mixed cellulose esters filters (Millipore, UK). MAIP S4510 plates were pre-wetted with 50 μl of 70% ethanol, washed five times with 200 μl of sterile phosphate buffered saline (PBS) and coated with 70 μl of 5 μg/ml anti-IFN-γ capture antibody, AN18 (Mabtech, Australia) in PBS and incubated at 4°C overnight (O/N). Seventy microliters of 5 μg/ml anti-IL-4 capture antibody (Mabtech, Australia) were added directly to MAHA S4510 plates and incubated at 4°C O/N without 70% ethanol treatment. Following five washes with PBS, plates were blocked by adding 200 μl of culture media (supplemented with 2.5% FCS) and incubated for 2 h at 37°C. The blocking media was discarded and 10 μg/ml recall peptides were added into each defined well. Con A (1.0 μg/ml) was used as internal positive control and no peptide (cells alone) as negative control. Triplicate wells were set up for each condition. About 5 × 10^5^ spleen cells in 100 μl culture media were seeded into each well and incubated at 37°C for 18 h (IFN-γ), or 24 h (IL-4), or 48 h for IL-10. Plates were washed five times with PBS/0.05% Tween 20 followed by five times with PBS and incubated for 2 h at RT with anti-murine IFN-γ, IL-4, or IL-10 monoclonal antibody-biotin. Plates were washed and streptavidin alkaline phosphatase (streptavidin–ALP) was added at 1.0 μg/ml and incubated for 2 h at RT. Spots of activity were detected using a colorimetric AP kit (Biorad, Hercules, CA USA) and counted using an AID ELISpot plate reader (Autoimmun Diagnostika GmbH, Germany). Data are presented as mean spot forming units (SFU) per 0.5 × 10^6^ cells ±SEM.

#### Proliferation

Spleen cells from immunized SJL/J mice were isolated 25–28 days after immunization and assessed by T cell proliferation assay. About 1 × 10^5^ spleen cells in 100 μl of culture media were seeded into 96 well *U*-bottom plates and incubated for 1–6 days at 37°C in the presence of recall peptide (10 μg/ml), ConA (internal control), or no peptide (negative control). ConA (internal positive control) yielded proliferation of more than 90,000 cpm and was excluded from the figures and no peptide (cells alone) was used as background negative control. Proliferation was assessed by adding 1 μCi of [^3^H]-thymidine per well to one plate per time point (days 1–6). Cells were incubated for 6 h before harvesting onto glass fiber filters. [^3^H]-thymidine uptake was measured using a β-scintillation counter (Top Count Gamma Counter, Packard, USA).

#### Statistical analysis

Mean values were compared using an unpaired *t*-test and one-way ANOVA. *p < 0.05 indicates a significant difference, **p < 0.001 indicates highly significant difference.

## Conflict of Interest Statement

Eldrug S.A., funded the project.
